# Feasibility of Mapping Austrian Health Claims Data to the OMOP Common Data Model

**DOI:** 10.1007/s10916-019-1436-9

**Published:** 2019-09-07

**Authors:** Andrea Haberson, Christoph Rinner, Alexander Schöberl, Walter Gall

**Affiliations:** Center for Medical Statistics, Informatics and Intelligent Systems, Medical University of Vienna, Spitalgasse 23, 1090 Vienna, Austria

**Keywords:** Standardized health data, Secondary use, Claims data, Drug safety, Common data model, OMOP

## Abstract

The Main Association of Austrian Social Security Institutions collects pseudonymized claims data from Austrian social security institutions and information about hospital stays in a database for research purposes. For new studies the same data are repeatedly reprocessed and it is difficult to compare different study results even though the data is already preprocessed and prepared in a proprietary data model. Based on a study on adverse drug events in relation to inappropriate medication in geriatric patients the suitability of the Observational Medical Outcomes Partnership (OMOP) common data model (CDM) is analyzed and data is transformed into the OMOP CDM. 1,023 (99.7%) of drug codes and 3,812 (99.2%) of diagnoses codes coincide with the OMOP vocabularies. The biggest obstacles are missing mappings for the *Local Vocabularies* like the Austrian pharmaceutical registration numbers and the Socio-Economic Index to the OMOP vocabularies. OMOP CDM is a promising approach for the standardization of Austrian claims data. In the long run, the benefits of standardization and reproducibility of research should outweigh this initial drawback.

## Introduction

The joint analysis of large medical data sources, such as electronic health records, claims data or routine data, is essential in order to compete and participate in current scientific research efforts. Many countries provide such health data in special databases for scientific research purposes, such as the Premier Perspective Database^1^ in the US or the DaTraV database [1] in Germany. In Austria, the Main Association of Austrian Social Security Institutions brings together claims data from various social security institutions and accounting data from the Minimal Basic Data Set with information about hospital stays in the database GAP-DRG (General Approach for Patient-oriented Ambulant Diagnosis Related Groups). The GAP-DRG is based on a proprietary data model and is available for research projects on health care issues.

The use of claims data for health analysis is an application of secondary use of data since the data were originally collected for a different purpose [2]. In the course of data preparation, assumptions have to be made under consideration of the research question concerning the data, which are often not sufficiently communicated and documented [3]. Also, in the case of the Austrian health claims data the same data are repeatedly reprocessed for different studies making it difficult to objectively compare study results even if they originate from the same database. Data standardization using a common data model (CDM) ensures syntactic and semantic interoperability, making studies comparable, analysis methods reusable and reduces the need to reprocess the same data multiple times. Standardized data increases data quality, enables the use of common query tools and reduces expenses in terms of costs and time.

The Observational Medical Outcomes Partnership (OMOP) CDM was originally developed to support drug safety. Furthermore, it is continuously improved by the international, interdisciplinary Observational Health Data Sciences and Informatics (OHDSI) community^2^ and it provides a rich terminology [4]. Based on a drug safety study, this article evaluates the feasibility of the OMOP CDM for secondary use of Austrian health claims data.

## Methods

The project „Adverse drug events in relation to inappropriate medication of geriatric patients with renal insufficiency - a retrospective register-based cohort study” (ADE-PIM^3^) with data from the GAP-DRG is used as an application case. The source data of ADE-PIM is mapped and transformed into the OMOP CDM. The process is divided into the following steps:

- Vocabulary mapping, to map relevant classifications and terminologies to the OMOP CDM concepts
- Data tables mapping, to map each relevant attribute of a local data source to the proper column in the OMOP CDM
- Extract-Transform-Load (ETL) process to transform and load the local data into the OMOP CDM
- Validation of integrity and equivalence to the local source data

A notable feature of the OMOP CDM is the available comprehensive Standardized OMOP Terminology, including 81 vocabularies^2^, referred to as *OMOP-Vocabularies* in the following. Terminologies used in the source data, referred to as *Local Vocabularies* in the following, must be prepared in such a way that all relevant medical terms are linked to equivalent *Standard Concepts* and imported as new concepts into the OMOP CDM. The amount of effort needed for this process depends on the particular state of the *Local Vocabulary*. We distinguish the following states of *Local Vocabularies*:

- State 1 (S1): *Local Vocabulary* included in the *OMOP-Vocabularies* one-to-one.
- State 2 (S2): *Local Vocabulary* not included in the *OMOP-Vocabularies* one-to-one, but semantically equivalent concepts in the *OMOP-Vocabularies* exist.
- State 3 (S3): *Local Vocabulary* not included in the *OMOP-Vocabularies* one-to-one and no semantically equivalent concepts in the *OMOP-Vocabularies*.

The OHDSI tool Athena^4^ is used to find *Local Vocabularies* within the *OMOP-Vocabularies*. *Local Vocabularies* in S1 can be directly mapped. If a semantically equivalent concept exists in the *OMOP-Vocabularies* (S2), its *Standard Concept* can be adopted. The OHDSI tool USAGI^4^ was used to find potential appropriate concepts. If no equivalent OMOP concept exists (i.e. S3), new *Standard Concepts* have to be modeled according to the defined structure of the relevant domain (relationships and hierarchy) in coordination with the OHDSI community.

As part of the data table mapping for each relevant attribute in the local data source, the proper column in the appropriate OMOP CDM data table has to be identified. The local data source is scanned by the OHDSI tool WhiteRabbit^4^ to create a detailed report of the local tables, columns and data types. Based on this report the rules for mapping local data fields to proper OMOP CDM fields and type transformations were specified.

Legal requirements demand that the available claims data must be processed on the original server, hence the Extract-transform-load (ETL) process is performed using a PostgreSQL database. The OMOP CDM version 6.0 instance was installed in a dedicated schema in the same database as the local source data. A collection of the *OMOP-Vocabularies* was downloaded from the Athena^4^ repository. The prepared local codes were added to the OMOP CDM tables *Vocabulary*, *Concept* and *Concept_Relationship* referencing the proper OMOP *Standard Concepts*. Python scripts and SQL queries were developed to handle the ETL process according to the data table mapping. Source records of patients, medications, hospital stays and diagnoses are gathered in a temporary data structure before transforming it and writing it to the designated data fields in the OMOP CDM.

In the final step, software routines were developed to test the transformed data for integrity and equivalence to the source data and row counts of corresponding tables were compared. SQL queries were developed to gather information on patients, medications, hospital stays and diagnoses respectively from the OMOP CDM into temporary tables. The tables were then compared record-wise to the corresponding source tables. Descriptive statistical indicators were calculated from the transformed data and compared to original values to demonstrate the success of the ETL process.

## Results

The GAP-DRG covers about 95% of the Austrian population. The ADE-PIM cohort used in this study is limited to one Austrian province, contains 12,505 patients over the age of 70 years and covers 3,842 different ICD10 codes, 1,026 different ATC codes, 7,164 different drugs and 18 different Professional Groups involved in the prescription process. This corresponds to 0.15% of the Austrian population, 33.9% of ICD10 codes, 70.7% of ATC codes, 23.7% of Professional Groups used and 40.5% of drugs prescribed in Austrian claims data.

In Table 1, the results of the vocabulary mapping with the identified medical terms and public codes systems used in this study are summarized. Diagnoses are coded in the Austrian ICD10 version of the Federal Ministry for Social Security and Generations (ICD10-BMSG). Drugs are explicitly identified by the Austrian Pharmaceutical Registration Number (PRN) and refer to their active ingredients by ATC codes. Professional Groups and Socio-Economic Index (SEI) are not encoded using a public code system. Based on these medical terms, the OMOP domains *Condition*, *Procedure, Measurement, Observation, Drug* and *Provider Specialty* were derived.

**Table 1:** Identified relevant medical terms of the ADE-PIM study and the results of the vocabulary mapping

*ADE-PIM Local Vocabulary*		*OMOP CDM*		State of ADE-PIM Local Vocabulary
Medical term	Public code system	*OMOP-Vocabularies*	Domain	
Diagnoses	ICD10-BMSG	ICD10-WHO ICD10-CM	Condition/Procedure/Measurement/Observation	S1/S2
Drugs				
active ingredient	ATC	ATC-WHO	Drug	S1/S2
medicinal product	PRN	RxNorm RxNorm Extension		S2/S3
Professional Groups		NUCC Medicare Specialty	Provider Specialty	S2/S3
SEI		SNOMED-CT	Observation	S3

3,812 ICD10-BMSG codes (99.2%) match the ICD10 OMOP-Vocabularies. Most of them are in the version of the World Health Organization (ICD10-WHO). 31 of them coincide the American ICD10-Clinical Modification (ICD10-CM). The remaining 30 ICD10-BMSG codes (e.g. *M51.28*) are in stage S2. 1,023 ATC codes (99.7%) are in stage S1. The missing ATC codes (S2) originate from past vocabulary changes, e.g. in 2012, *D11AX19 alitretinoin* was altered into *D11AH04*. For the ICD10 and ATC codes in S2 equal concepts were found using the USAGI tool. From the 27 Professional Groups in the source data 4 could not be matched (e.g. *other facility*) (S3). The specific algorithm used in Austria to calculate the SEI is not comparable to any of the *Socioeconomic status* concepts in OMOP hence it is in S3. Finally, all *Local Vocabularies* in S1 and S2 were imported into the OMOP CDM as new concepts referencing their equivalent *Standard Concepts*. PRNs, Professional Groups and SEI in stage S3 were not imported in a standardized fashion yet.

**Fig. 1:**
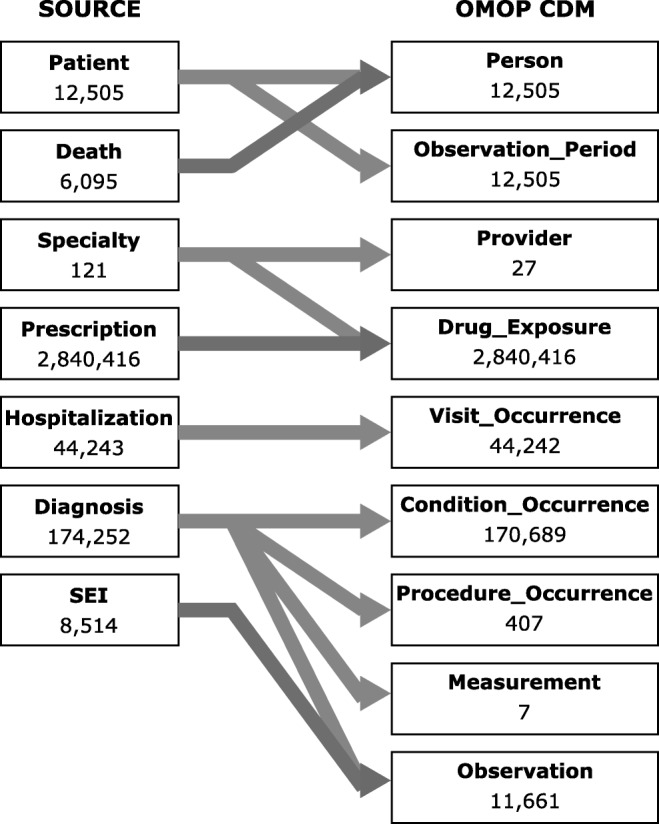
Data table mapping and number of rows per table before and after the ETL process. Duplicate records of hospitalizations and diagnoses were removed during ETL

Figure 1 provides an overview of the local data source and the data table mapping is given. Data from the tables with personal information (i.e. *Patient*) and death dates (i.e. *Death*) were combined in one table (i.e *Person*). The observation period was set to last the whole study period (i.e. 2008 to 2011) since no period was available in the source data. Other source data did not naturally map to the OMOP CDM. For example, no information on individual health care providers was available beside the medical specialty stated on prescriptions. The OMOP CDM, on the other hand, allows a provider to only have one single specialty, which would result in loss of relevant information. We solved this problem by replacing individual providers with virtual providers for each of the occurring specialties. Diagnosis data from the source had to be split across the tables *Condition_Occurrence*, *Procedure_Occurence*, *Measurement* and *Observation* to adhere to the standard. Some obligatory data fields are not available in the source data (e.g. race, ethnicity, gender of providers or the drug exposure end date). These fields had to be filled with insignificant data (i.e. the OMOP specification recommends to use the *concept id* 0 in this case).

Running the validation scripts indicated that the ETL process was successful and no records were lost. The distribution of sex (50.82% female), age (median: 78) as well as the number of hospitalizations per person (median: 2), number of prescriptions (median: 192) and length of hospitalization (median: 5 days) were equal to the source data.

## Discussion

In a prior study we implemented a clinical data warehouse based on the OMOP CDM [5]. Results like the ETL process were reused in this study. This current study extends the work insofar as the vocabularies used in Austrian claims data were analysed in more detail and real data from the ADE-PIM study were transformed into the OMOP CDM. The transformation of existing data into the OMOP CDM is a complex process requiring knowledge of the source data as well as in-depth knowledge of the OMOP CDM.

Although the ADE-PIM relevant data are 0.15% of the Austrian population, they represent 33.9% of ICD10 codes and 70.7% of ATC codes used in Austrian claims data. Most of these codes are in the *OMOP-Vocabularies* (S1), hence they demand the least mapping effort. The preparation of terms in S2 (e.g. Professional Groups) and S3 (PRN) is more complex. Mappings of S2 terms were done by a medical informatics student and S3 terms exceeds the projects ressources. As all OMOP analysis tools are based on the OMOP *Standard Concepts*, in future studies domain experts with adequate time resources, profound technical knowledge and a basic understanding of the structure of each OMOP domain will be needed to ensure an optimal mapping outcome. Since the ADE-PIM project includes only few domains, 15 OMOP data tables and attributes are empty (e.g. no costs, no locations, etc.). To gather more experience additional studies on the GAP-DRG, even already completed ones, should be transformed into OMOP.

An outstanding advantage of standardized data are the use of common query tools ensuring comparability of studies, the reusability of methods and the reduction of expenses in terms of costs and time. All OMOP analysis tools are based on the OMOP *Standard Concepts* (i.e. for diagnosis SNOMED-CT instead of ICD10). Thus, further studies have to be conducted evaluating the handling of these tools in practice.

Our results are comparable to studies in other countries. For example, Kim et.al [6] explored the feasibility to import nursing data from the US into OMOP and Maier et.al. [7] mapped the vocabularies to import the data of hospitals in Germany. In both studies it is concluded that OMOP provide good representation for a majority of the data, but also gaps resulting to information loss or additional effort are reported.

The question of whether the OMOP CDM is a feasible model to Austrian health claims data can not be answered at this stage. Further investigations are necessary, on the one hand transforming additional medical terms, on the other hand evaluating the handling of OMOP analysis tools based on transformed Austrian health claims data. Based on the presented study results the OMOP CDM is a promising approach for the standardization of Austrian claims data and only one-off resource consuming. In the long run, the benefits of standardization and reproducibility of research should outweigh this initial drawback.

Based on our experience we plan to revise the vocabulary mapping of S2 terms and integrate missing *Standard Concepts* (S3 terms) of relevant ADE-PIM data. This process is planned to take place in consultation with the OHDSI community, intending to publish the new concepts in the OMOP CDM making them available for future studies. In a next step, the ADE-PIM cohort will be built based on the *Standard Concepts* of the transformed data and the performance of OMOP query tools will be evaluated. All these steps will help answering the question whether OMOP is an appropriate way to standardize Austrian health claims data.

SNOMED-CT is used as *Standard Concept* in OMOP. Since the beginning of 2019, Austria is a member of SNOMED International and SNOMED-CT can now be officially used to encode Austrian medical information. In this project, we used claims data from 2008 to 2011. If and when SNOMED-CT mappings are available for Austrian claims data is uncertain, yet it is an additional step towards internationally harmonized reproducible research.

## Footnotes

1 Premier Applied Sciences® (2018) Premier Healthcare Database White Paper: Data that informs and performs. Premier Inc. https://www.premierinc.com/downloads/PremierHealthcareDatabaseWhitepaper.pdf. Accessed 14 May 2019

2 OHDSI (2019) Observational Health Data Sciences and Informatics https://ohdsi.org/. Accessed 5 March 2019

3 Project website ADE-PIM study, MedUni Vienna https://cemsiis.meduniwien.ac.at/en/mim/sr/projects/current/ade-pim/. Accessed 27 May 2019

4 OHDSI (2019) Open-Source Software on GitHub Repository. GitHub, Inc. https://github.com/OHDSI/. Accessed 27 May 2019
